# Epidemiology and bacterial characteristics of invasive group B streptococcus disease: a population-based study in Japan in 2010–2020

**DOI:** 10.1017/S0950268822001534

**Published:** 2022-10-07

**Authors:** Noriko Takeuchi, Bin Chang, Kenichi Takeshita, Sachiko Naito, Yoshiko Takahashi, Haruka Hishiki, Naruhiko Ishiwada

**Affiliations:** 1 Department of Infectious Diseases, Medical Mycology Research Center, Chiba University, Chiba, Japan; 2 Department of Bacteriology I, National Institute of Infectious Diseases, Tokyo, Japan; 3 Department of Pediatrics, Chiba University Graduate School of Medicine, Chiba, Japan

**Keywords:** Group B streptococcus, invasive group B streptococcus disease, MLST, serotype, *Streptococcus agalactiae*

## Abstract

This is the first report on a population-based prospective study of invasive group B streptococcus (GBS) disease among children aged <15 years conducted over a period of 11 years in Japan. This study investigated the incidence and clinical manifestations of invasive GBS disease in children in Chiba Prefecture, Japan, and analysed the serotypes and drug susceptibility of GBS strains isolated during the study period. Overall, 127 episodes of invasive GBS disease were reported in 123 patients. Of these, 124 were observed in 120 patients aged <1 year, and the remaining three episodes were reported in a 9-year-old child and two 14-year-old children with underlying disease. For patients aged <1 year, the incidence rate per 1000 live births was 0.24 (0.15–0.36). The incidences of early-onset disease and late-onset disease were 0.04 (0.0–0.09) and 0.17 (0.08–0.25), respectively. The rate of meningitis was 45.2%, and the incidence of GBS meningitis was higher than that of other invasive diseases among children in Japan. Of the 109 patients for whom prognosis was available, 7 (6.4%) died and 21 (19.3%) had sequelae. In total, 68 strains were analysed. The most common were serotype III strains (*n* = 42, 61.8%), especially serotype III/ST17 strains (*n* = 22, 32.4%). This study showed that the incidence of invasive GBS disease among Japanese children was constant during the study period. Because of the high incidence of meningitis and disease burden, new preventive strategies, such as GBS vaccine, are essential.

## Introduction

*Streptococcus agalactiae* (group B streptococcus (GBS)) are Gram-positive bacteria. GBS infection can cause invasive disease and lead to severe life-threatening conditions in infants, especially neonates [1]. Therefore, preventing invasive GBS disease in neonates is one of the most urgent global medical issues. Invasive GBS disease in infants is classified based on the time of onset as early-onset disease (EOD; onset within the first 6 days of life), late-onset disease (LOD; onset from 7 to 89 days of life) and late late-onset disease (LLOD; occurs in infants aged >3 months) [2, 3]. The incidence of EOD can be reduced by preventing vertical infection through intrapartum antimicrobial prophylaxis (IAP) based on maternal universal screening results at 35–37 weeks of gestation [2]. However, IAP is not effective in preventing LOD [4]. As a preventive measure for LOD, several capsular serotype-specific GBS vaccines have been recently developed [5]. Clinical trials of these vaccines have been conducted, and they are expected to be effective [6].

Accurate up-to-date information on the incidence of invasive GBS disease is fundamental in estimating vaccine efficacy and decision-making for GBS vaccine management as applicable country wise. However, limited information is available regarding the population-based incidence of invasive GBS disease among children in Japan [7–9]. In this study, the incidence and clinical manifestations of invasive GBS disease in children in Chiba Prefecture, Japan, were investigated and the serotypes and drug susceptibility of isolated GBS strains during the study period of 11 years analysed.

## Methods

### Incidence and clinical information of invasive GBS disease

To determine the precise incidence of invasive GBS disease in Chiba Prefecture, Japan, we applied the reporting system for invasive GBS disease in all hospitals with newborn and paediatric wards in Chiba Prefecture and those in the areas surrounding Chiba Prefecture. This prefecture is one of the 47 prefectures in Japan that is located in central Japan. Chiba Prefecture has a population of approximately 6 million people, accounting for 5% of the total population of Japan. When treating children with invasive GBS disease, physicians in the hospital use standardised case report forms to record patient and laboratory information and then send the form to Chiba University through fax or e-mail. In addition to immediate reporting when a patient was hospitalised, a survey was conducted twice a year to ensure that there are no omissions. This active surveillance study was initiated in January 2008, and data from January 2010 to December 2020 were analysed. Invasive GBS disease was defined as the detection of GBS infection in the blood or cerebrospinal fluid. Cases in which blood culture was positive and meningitis was suspected based on clinical symptoms, such as convulsive seizures, but cerebrospinal fluid could not be examined were classified as meningitis. According to the time of onset, we classified invasive GBS disease as EOD (onset within the first 6 days of life), LOD (onset from 7 to 89 days of life) and LLOD (occuring in infants aged >3 months) [2, 3].

For each case, the clinical background, such as patient age, underlying diseases, onset, maternal carriage status, maternal IAP administration, treatment and prognosis was confirmed. We selected only patients who lived in Chiba Prefecture. The incidence rate per 1000 live births for patients aged <1 year was determined. Furthermore, the incident rate per 100 000 population aged <5 years was determined for comparison with the incidence of other invasive diseases. The incidence of invasive GBS disease per 1000 live births was calculated based on the population by age in Chiba Prefecture on 1st April of the next year as live births of the year [10]. The incidence rate per 100 000 population aged <5 years was calculated using the database on the same website [10]. We also conducted a survey on maternal screening culture sites and methods since 2016.

### Capsular serotyping and multi-locus sequence typing

The isolated strains were sent to Chiba University and serotypes were analysed. The serotypes were determined using the slide agglutination method by utilising the *S. agalactiae* antisera ‘Seiken’ set (Denka Seiken, Tokyo, Japan) and polymerase chain reaction (PCR) [11]. All collected strains were sent to the National Institute of Infectious Diseases where the serotype was confirmed using the same methods. Moreover, molecular typing was performed by employing multi-locus sequence typing (MLST) to analyse seven housekeeping genes (*adhP*, *pheS*, *atr*, *glnA*, *sdhA*, *glcK* and *tkt*) by PCR, as previously described [12]. PCR products were purified using a FastGene Gel/PCR Extraction Kit (NIPPON Genetics) and subjected to sequence analysis with the BigDye Terminator version 3.1 Cycle Sequencing Kit on an Applied Biosystems 3130xl Genetic Analyser (Applied Biosystems). Allelic numbers, sequence types (STs) and clonal complexes (CCs) were determined using an MLST database [13].

### Antimicrobial susceptibility test

Antimicrobial susceptibilities to penicillin G, ampicillin, cefotaxime, meropenem, erythromycin, clindamycin, ciprofloxacin and vancomycin were examined using the broth microdilution method according to the Clinical and Laboratory Standards Institute (CLSI) standard method, and the minimum inhibitory concentration (MIC) was determined [14].

### Statistical analyses

The *χ*^2^ test was used for the statistical analysis. Statistical significance was set at a *P-*value of <0.05.

### Ethical considerations

This study was approved by the Chiba University Ethics Committee (approval no. 666).

The authors assert that all procedures contributing to this work comply with the ethical standards of the relevant national and institutional committees on human experimentation and with the Helsinki Declaration of 1975, as revised in 2008.

## Results

### Annual number and incidence of invasive GBS disease

**Fig. 1.**
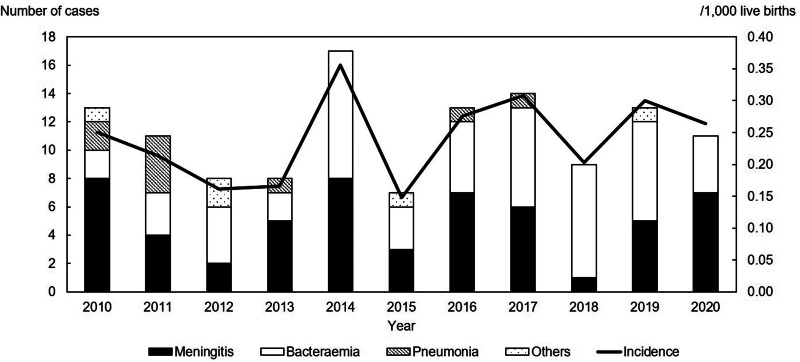
Annual number and incidence of invasive GBS disease among infants in Chiba, Japan. The annual number and the incidence rate per 1000 live births ranged from 7 to 17 and 0.15 to 0.36 for the 11-year period of 2010–2020.

Figure 1 shows the annual number and incidence of invasive GBS disease per 1000 live births. A total of 127 invasive GBS disease episodes were reported in 123 patients in the 11-year period from 2010 to 2020. Of the 127 episodes, 124 were observed in patients aged <1 year (120 patients), whereas the remaining three episodes were observed in one patient aged 9 years and two patients aged 14 years. Four patients (3.3%) had recurrent invasive GBS disease. The annual number and incidence rate per 1000 live births were 7–17 and 0.15–0.36, with an average value of 11.3 and 0.24, respectively.

In Japan, GBS screening of pregnant women and IAP for GBS-positive mothers were recommended by the Japanese Society of Obstetrics and Gynaecology in 2008. Until 2017, these guidelines recommended GBS culture testing at 33–37 weeks of gestation. The 2017 revision recommended that the GBS culture test be performed at 35–37 weeks of gestation [15]. The incidence of invasive GBS disease per 1000 live births is not statistically different between the pre-revision (2010–2016) and post-revision (2017–2020) periods (*P* = 0.431).

Supplementary Table S1 shows the yearly changes in the types of invasive GBS diseases in patients aged <1 year. Of all 124 cases, 23 were EOD, 86 were LOD and 10 were LLOD cases. All five cases that could not be classified based on the time of onset were observed in infants aged <1 month. The four cases of recurrences were LOD. The average incidences of EOD, LOD and LLOD were 0.04 per 1000 live births (range, 0.0–0.09), 0.17 per 1000 live births (range, 0.08–0.25) and 0.02 per 1000 live births (range, 0.00–0.08), respectively. The incidence of EOD and LOD is not statistically different for the pre- and post-revision periods (*P* = 0.981 and *P* = 0.060). Furthermore, the annual incidence of meningitis due to invasive GBS infection was 0.02–0.17 per 1000 live births, with an average value of 0.11 per 1000 live births. Annually, meningitis accounted for 11.1%–63.6% of all cases in those aged <1 year and 45.2% overall. According to the time of onset, 43.5% (10/23) of meningitis cases were EOD, 47.7% (41/86) were LOD and 50.0% (5/10) were LLOD, with no significant differences (*P* = 0.92).

Conversely, the average incidence of invasive GBS disease was 4.51 per 100 000 population aged <5 years (range, 2.81–6.72), and the average incidence of meningitis was 2.04 per 100 000 population aged <5 years (range, 0.42–3.16).

### Background information of the cases with invasive GBS disease

Table S2 shows the background information of patients aged <1 year with invasive GBS disease. Information on underlying diseases was available for 114 of 120 patients. Of the 40 patients with underlying diseases, 38 were premature babies in terms of preterm birth or low birth weight. Regarding the clinical diagnoses of 124 episodes, 54 patients had bacteraemia, 56 had meningitis, 9 had pneumonia and 5 had other forms of invasive disease. Among the four cases of recurrence, three had recurrent bacteraemia and one had been initially treated for bacteraemia but had cellulitis at the time of recurrence of bacteraemia. In all four cases, the first and second onsets were within 1 month.

Invasive GBS disease was observed in a pair of twins, with both being affected. Each of the twins developed bacteraemia at 21 days and 74 days, respectively, and the interval was 53 days. In three cases where only one of the twins had invasive GBS disease, each one had meningitis at 8 days of age, bacteraemia at 1 month of age and meningitis at 4 months of age, respectively. The co-twin of the last case died in utero. All five cases were in-hospital cases that were admitted to the neonatal intensive care unit after birth for preterm very low birth weight or very low birth weight.

Of the three patients aged ≥1 year, one was a 9-year-old with bacteraemia after bone marrow transplantation for aplastic anaemia, one was a 14-year-old who had bacteraemia with rhabdomyosarcoma as the underlying disease, and one was a 14-year-old who had bacteraemia with cellulitis, left heart hypoplasia and protein leakage gastroenteropathy as underlying diseases.

### Treatment and prognosis of patients with invasive GBS disease

More than 10 antimicrobial regimens were selected for the treatment (Table 1). The regimen involving initial antibiotic treatment with ampicillin or sulbactam/ampicillin and cefotaxime or ceftriaxone was the most common and used in 62 episodes; in addition to the two drugs, aminoglycosides such as gentamicin, tobramycin, amikacin and arbekacin were administered in six episodes. The regimen of ampicillin or sulbactam/ampicillin combined with antimicrobial agents other than cefotaxime or ceftriaxone was applied in 23 episodes, 21 of which included combinations with an aminoglycoside. Ampicillin alone was used in 16 episodes. Steroids were used in 22 episodes, and intravenous human immunoglobulin was used in five episodes. Table 1 shows the prognosis of patients aged <1 year with invasive GBS disease in this study. Among the 124 episodes, 43 had some complications such as intracranial haemorrhage, subdural oedema, septic shock and convulsions. The prognosis of 109 patients was available: 81 (75.0%) completely recovered, 21 (19.3%) had sequelae and 7 (6.4%) died. Most sequelae were neurological sequelae such as developmental delay/intellectual disability, motor deficit, vision impairment, hearing impairment, epilepsy, hypertonia, cerebral palsy and one case was bone deformity associated with arthritis. No significant differences were observed in terms of prognosis and onset time, or prognosis and meningitis. We also investigated the correlation between steroid use and known prognosis of meningeal inflammation in 51 cases. Of the 16 patients who used steroids, 3 died, 3 had sequelae and 10 completely recovered. In contrast, among 35 patients who did not use steroids, 2 died, 12 had sequelae and 21 completely recovered. No significant difference was observed in the prognosis because of steroid use.

**Table 1.** Antimicrobial treatment and prognosis of patients with invasive GBS disease aged <1 year (*n* = 124)

	Number of cases (%)
Antimicrobial treatment	
AMP + CTX	51 (41.1)
AMP	14 (11.3)
AMP + GEN	8 (6.5)
AMP + AMK	5 (4.0)
AMP + TOB	5 (4.0)
PAB + CRO	5 (4.0)
AMP + CTX + GEN	3 (2.4)
CTX	3 (2.4)
SAM	3 (2.4)
AMP + CTX + AMK	2 (1.6)
AMP + CRO	2 (1.6)
Others	19 (15.3)
Unknown	3 (2.4)
Unused	1 (0.8)
Use of steroid	
Yes	22 (17.7)
No	98 (79.0)
Unknown	4 (3.2)
Complications	
Yes	47 (37.9)
No	70 (56.5)
Unknown	7 (5.6)
Prognosis^a^	
Cure	81 (67.5)
Sequelae	21 (17.5)
Death	7 (5.8)
Unknown	11 (9.2)

AMP, ampicillin; AMK, amikacin; CTX, cefotaxime; CRO, ceftriaxone; GEN, gentamicin; PAB, panipenem/betamipron; SAM, sulbactam/ampicillin; TOB, tobramycin.

a Recurrent cases were counted only once.

### Maternal information of invasive GBS disease

**Fig. 2.**
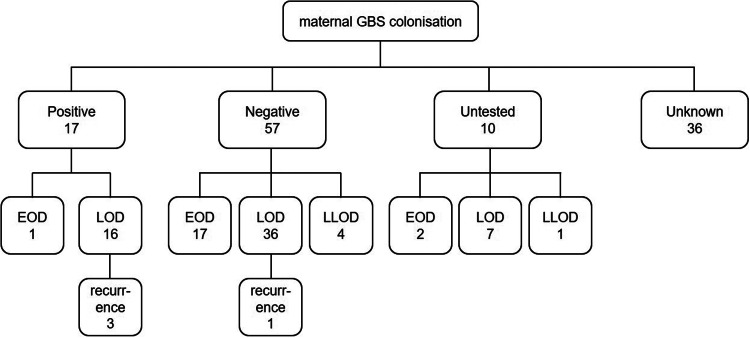
Maternal GBS colonisation and invasive GBS disease in infants. Maternal GBS colonisation status was available for 74 patients. EOD, early-onset disease; LOD, late-onset disease; LLOD, late late-onset disease.

Figure 2 and Supplementary Table S3 show the maternal information of patients aged <1 year with invasive GBS diseases. Of the 120 patients, information on the mode of delivery was available for 98 patients, and 72 patients (73.5%) were born through vaginal delivery. Maternal GBS colonisation status was available for 74 patients, and the status was positive in 17 (23.0%) patients. Of the five patients tested for maternal GBS colonisation who were born before 35 weeks of gestation, one showed positive result. There were also 10 untested cases, and eight patients were born before 35 weeks. Information on maternal IAP status was available for 85 patients, and 22 (25.9%) mothers of patients had received antimicrobials. Of the 17 maternally positive cases, 3 were unknown for IAP, and all others received IAP. Information on the nutrition status of the patients was available for 95 patients, and 45 patients (47.4%) had mix nutrition. Among the 23 patients with EOD, maternal GBS colonisation was positive in only one case whose mother received antimicrobials during delivery, whereas 17 cases were negative for maternal GBS colonisation at the time of screening. In the four cases of recurrence, maternal screening during pregnancy was positive in three cases and negative in one case. Of the three positive mothers, two received IAP, and one was prescribed an oral antimicrobial for eradication during pregnancy, although it was unclear whether she had received IAP. All patients received mixed nutrition or were breastfed. If invasive GBS disease recurred in the infant, the mother's milk was cultured, and the mother was treated with antimicrobials regardless of the culture outcome. GBS was detected in the milk of one patient who was positive and one patient who was negative in the screening of pregnant mothers. In both cases, the mother-derived GBS, serotype and ST were the same as the infant isolate, and breastfeeding was discontinued.

### Antimicrobial susceptibility testing of isolated strains

Overall, 68 strains were preserved and collected in Chiba University. Table 2 shows the results of the antimicrobial susceptibility testing for 68 strains. All tested strains were susceptible to penicillin. The MIC90 and MIC50 values of penicillin G were 0.06 μg/ml each. The MIC90 values of ampicillin, cefotaxime, meropenem and vancomycin all fell in the susceptible category, whereas the MIC90 values of erythromycin and clindamycin fell in the resistant category, and the rates of resistant isolates were 57.4% (39/68) and 29.4% (20/68), respectively. The MIC90 and MIC50 values of ciprofloxacin were 2 μg/ml and 1 μg/ml, and 6 isolates had MIC values of ciprofloxacin >16 μg/ml. Of the remaining 59 strains that could not be examined, we were able to collect the drug susceptibility testing data for 47 of 59 strains from each hospital where the GBS strain was isolated; all 47 were susceptible to penicillin.

**Table 2.** Antimicrobial susceptibility testing of isolated strains (*n* = 68)

Name of antimicrobials	Susceptibility break points^a^	MIC range	MIC 50	MIC 90	Resistance rates	Sensitivity rates
PEN	MIC ≤ 0.12	0.03–0.12	0.06	0.06	0%	100%
AMP	MIC ≤ 0.25	0.03–0.25	0.12	0.12	0%	100%
CTX	MIC ≤ 0.5	0.03–0.12	0.06	0.06	0%	100%
MEM	MIC ≤ 0.5	0.015–2	0.03	0.06	0%	100%
ERY	MIC ≤ 0.25	0.12–>8	1	>8	57.4%	41.2%
CLI	MIC ≤ 0.25	0.12–>8	0.25	>8	29.4%	66.2%
CIP	Not determined	0.5–>16	1	2	Not determined	Not determined
VAN	MIC ≤ 1	0.5	0.5	0.5	0%	100%

MIC, minimum inhibitory concentration, PEN, penicillin G; AMP, ampicillin; CTX, cefotaxime; MEM, meropenem; ERY, erythromycin; CLI, clindamycin; CIP, ciprofloxacin; VAN, vancomycin.

MIC values are presented in μg/ml.

a MIC breakpoints by the CLSI. MIC breakpoints for CPFX are not determined.

### Capsular serotyping and MLST of isolated strains

**Fig. 3.**
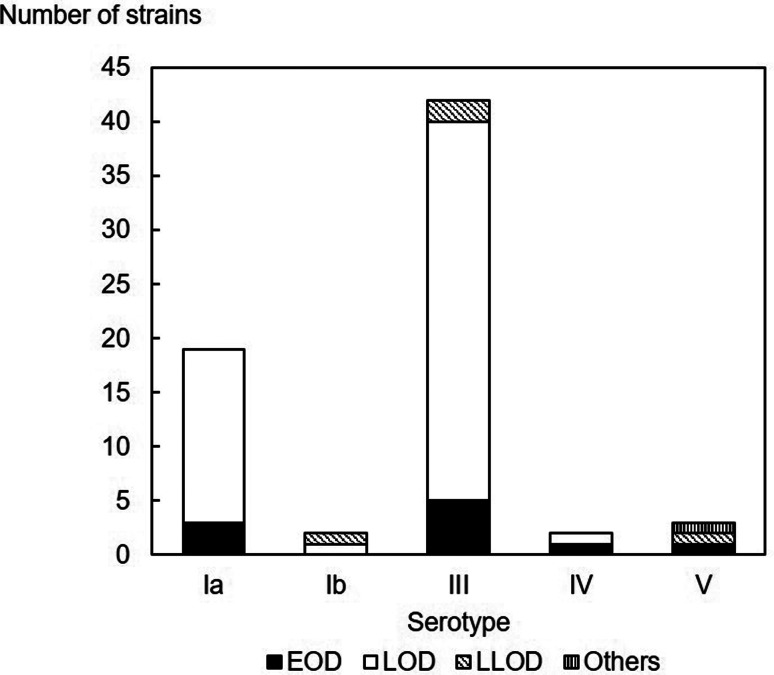
Serotype distribution of the isolated strains (*n* = 68). Overall, 68 isolates were classified into five serotypes: Ia, Ib, III, IV and V. Serotype III was 61.8% (42/68), serotype Ia was 27.9% (19/68), and serotypes V, Ib, and IV were 4.4% (3/68), 2.9% (2/68), and 2.9% (2/68), respectively. EOD, early-onset disease; LOD, late-onset disease; LLOD, late late-onset disease.

Regarding capsular serotyping, 68 isolates of 127 episodes were tested and classified into five serotypes: Ia, Ib, III, IV and V (Fig. 3). Serotype III was the most frequent with 61.8% (42/68), followed by serotype Ia with 27.9% (19/68) and serotypes V, Ib and IV with 4.4% (3/68), 2.9% (2/68) and 2.9% (2/68), respectively. Regarding sequence typing, 68 isolates were tested and classified into 13 STs and 6 CCs (Table 3). The most frequent ST was ST17 of serotype III (22 isolates), followed by ST23 of serotype Ia, and serotype III (serotype Ia, 16 isolates; serotype III, 1 isolate). Among the 22 serotype III/ST17 strains, 13 (59.0%) strains were resistant to both erythromycin and clindamycin. Of the four cases of recurrent invasive GBS disease, the strain was not preserved at the time of initial onset in one case, and only the strain at the time of recurrence was subjected to bacteriological examination. In the remaining three cases, the serotype and ST at the first onset and at the time of recurrence were consistent. All four cases had serotype III/ST17. Of the three cases in patients aged ≥1 year, only one had a detected serotype and ST, and serotype V/ST26 strain was derived from the 14-year-old patient with bacteraemia and cellulitis.

**Table 3.** Characteristics of GBS isolates (*n* = 68)

		Serotype					Antimicrobial resistance				
CC (no. of isolates)	ST	Ia	Ib	III	IV	V	PEN	AMP	CTX	ERY	CLI
CC17 (22)	ST17 (22)	0	0	22	0	0	0	0	0	13	13
CC23 (21)	ST23 (17)	16	0	1	0	0	0	0	0	9	1
	ST24 (2)	2	0	0	0	0	0	0	0	0	0
	ST452 (1)	0	0	0	1	0	0	0	0	0	0
	ST498 (1)	1	0	0	0	0	0	0	0	0	0
CC19 (19)	ST335 (10)	0	0	10	0	0	0	0	0	10	3
	ST27 (5)	0	0	5	0	0	0	0	0	4	3
	ST19 (3)	0	0	3	0	0	0	0	0	0	1
	ST575 (1)	0	0	1	0	0	0	0	0	1	0
CC10 (3)	ST10 (2)	0	1	0	1	0	0	0	0	1	1
	ST654 (1)	0	1	0	0	0	0	0	0	0	0
CC1 (2)	ST1 (2)	0	0	0	0	2	0	0	0	1	1
CC26 (1)	ST26 (1)	0	0	0	0	1	0	0	0	0	0
Total (%)		19 (27.9)	2 (2.9)	42 (61.8)	2 (2.9)	3 (4.4)	0	0	0	39	23

CC, clonal complex; ST, sequence type; PEN, penicillin G; AMP, ampicillin; CTX, cefotaxime; ERY, erythromycin; CLI, clindamycin.

## Discussion

In Japan, notification under the Infectious Diseases Control Law is not obligatory for invasive GBS disease. Although there are several surveys based on questionnaires, they were reports based on the number of births at the target facilities, and the population-based incidence was not accurately reported. This is the first report on a prospective population-based study of invasive GBS disease among children aged <15 years in Japan.

No particular tendency for fluctuations in the incidence of invasive GBS disease among children aged <1 year has been observed. The incidence rate of per 1000 live births was higher than the incidence rate of surveillance in 10 prefectures in Japan reported by Chang *et al*. from 2007 to 2012 [7]. In contrast, it was lower than the incidence rate reported by a nationwide surveillance of 343 hospitals in Japan in 2016–2020 [9]. The incidence of the present survey was different from those previous studies because of the differences of survey period, survey area and consideration of hospitalised cases in neighbouring prefectures. The survey period overlapped with that of the nationwide retrospective questionnaire survey by Shibata *et al*., but there was no mention of the participating institutions, and it is unclear how much of our data were included. Moreover, reports including the number of births per facility may have a selection bias for the target facility. Both EOD and LOD incidence rates per 1000 live births were lower than those in the United States, which is implementing IAP as in Japan [16,17].

In this study, we also calculated the incidence rate per 100 000 population aged <5 years for comparison with other invasive diseases. The incidence of invasive GBS disease per 100 000 population aged <5 years was 4.51 (range, 2.81–6.72) and that of meningitis was 2.04 (range, 0.42–3.16). Previous reports have shown that the incidence rate of invasive *Haemophilus influenzae* and invasive pneumococcal disease per 100 000 population aged <5 years after the introduction of the *Haemophilus influenzae* type b vaccine and pneumococcal conjugate vaccine in Japan were <1.0 and approximately 10, respectively. The incidence of meningitis per 100 000 population aged <5 years were approximately 0 and <1.0, respectively [18,19]. Compared with these, invasive GBS disease has a particularly high incidence of meningitis. Furthermore, our study found extremely few cases of invasive GBS disease in children aged ≥1 year.

Focusing on the bacterial profile, the most common serotype in this study was serotype III, which was the same as that reported in other domestic studies and studies conducted in other countries [9, 16, 20, 21]. Furthermore, the proportion of ST17, recognised as a hypervirulent international clone associated with invasive neonatal disease, was as high as 29.7% in 19 of the 64 patients aged <1 year in whom ST could be identified. In addition, serotype III/ST17 reportedly is largely capable of crossing the blood–brain barrier and is highly associated with meningitis; however, there was no significant association between serotype III/ST17 and meningitis in this study [22].

The rate of recurrence was 3.3% in this study, which was consistent with those in previous reports of 2.9% and 3.7% in Japan [8, 9]. Interestingly, all four cases of recurrence were caused by serotype III/ST17, and two recurrence cases were positive in breast milk culture. All GBS derived from breast milk belonged to serotype III/ST17; moreover, breast milk was suspected to be involved in the recurrence. However, it was unclear whether the infection was first established in the infant due to contamination of the infant's milk or due to a retrograde infection from the infant's oral cavity to the breast milk. In the review by Zimmermann *et al*. on LOD due to breast milk infection, 19 of the 59 cases (32%) had one or more recurrences, which is clearly higher than the recurrence rate (0.5%–3%) in general LOD. Moreover, the serotype in 22 of the 30 cases of infant–mother pairs was serotype III, and serotype III/ST17 strains were found in 5 cases, 3 of which were recurrent [23]. However, no consensus on the prevention and management of breast milk-associated LOD has yet been established [24].

Regarding drug susceptibility, penicillin- and cefotaxime-resistant strains have been reported in non-pregnant adults in Japan but not in the paediatric population [25]. However, penicillin-susceptible, ceftibuten-low susceptible isolates harbouring amino acid substitutions in PBP2X were reported in pregnant women in 2019 [26]. In this study, no penicillin- or cefotaxime-resistant strains were found.

In this study, the positive rate of maternal screening in EOD was 5.0% (1/20), and the negative rate of maternal screening in EOD was 85.0% (17/20). The low positivity rate of maternal screening in EOD is possibly because of the effectiveness of maternal IAP in preventing EOD in infants. On the other hand, the high incidence of EOD despite obtaining negative results on maternal screening may be because of improper specimen collection and culture methods for maternal screening. The current Japanese guidelines recommend culturing vaginal–rectal specimens obtained at 35–37 weeks of gestation using selective agar media. However, in Japan, the compliance rate of the guidelines is reportedly low with regards to the weeks of gestation, specimens and medium [27]. In this study, regarding the site of maternal screening, only 22 of 60 cases aged <1 year since 2016 were clear, of which only two cases were vaginal–rectal cultures that comply with the guidelines; 19 cases were vaginal cultures only, and two cases were vaginal-perianal cultures. Moreover, for cases in which vaginal–rectal cultures were performed, infants developed EOD despite receiving negative maternal culture results. Regarding the medium used for culturing, the results of only 11 cases were clear, among which a selective medium was used in two cases and a non-selective medium was used in nine cases. Ensuring proper sampling, culturing and identification is important to further increase the prevention of EOD.

Regarding LOD, of the 20 maternal GBS-positive cases (17 patients), 19 cases (16 patients) were LOD, and the positive rate of maternal screening among the LOD cases was 27.1% (16/59) in this study. Of the 16 positive mothers in LOD, 13 received IAP, except for 3 who uncertain of receiving IAP. This indicates that IAP is not effective in preventing LOD. Shibata *et al*. reported that neonatal GBS colonisation at 1 month after birth was higher than that at birth and 1 week after birth, and the GBS isolated was of the same serotype and ST as that which colonised the mother before delivery. It is suspected that after IAP was initiated, the density of maternal GBS colonisation decreased temporarily, but small numbers of GBS were transmitted from mother to baby and may gradually occur in the respiratory or intestinal tract [28]. The source of LOD is not limited to the mother but may be caused by horizontal transmission from other than mother after birth. However, the positive rate of maternal screening in LOD is not low, and detection of the serotypes and STs of the maternal GBS before delivery may clarify the transmission route in LOD. In contrast, focusing on the case of twins in this study, only one of the five cases had invasive GBS disease in both twins, but only one of the twins was infected in the remaining three cases. Of these, the maternal screening results were clear in two cases, both of which were negative. In such cases, examining the GBS carrier status of the twin who does not have invasive GBS disease may help determine the route of transmission.

Invasive GBS disease has a high incidence of mortality and sequelae and cannot be underestimated. Therefore, a maternal GBS vaccine has been developed, clinical trials have been conducted, and it is expected to be effective. In the future, to introduce this GBS vaccine in Japan, population-based incidence, disease burden and serotype distribution of invasive GBS disease will be required. In this study, invasive GBS disease caused by serotypes Ia, Ib and III, contained in the trivalent vaccine currently under development, was noted in 92.6% (63/68) of the cases, and effectiveness of the vaccine can be expected. However, capsular switching, such as in *Streptococcus pneumoniae*, may increase invasive pneumococcal disease with other serotypes after polysaccharide vaccine introduction. In fact, capsular switching has already been reported in serotype III/ST17 [29]. Therefore, considering whether only these serotypes should be targeted is essential. Another problem with vaccines with respect to invasive GBS disease is that it is necessary to acquire maternal anti-GBS antibodies in the second trimester of pregnancy in prematurely low birth weight infants, whereas the transferred maternal anti-GBS antibodies need to be maintained until the age of 1 year in infants. Thus, further clinical studies on the vaccine are warranted. Furthermore, in Japan, it is essential to develop a domestic active surveillance system to verify the effects of the introduction of the vaccine before considering the actual introduction.

There are several limitations in this study. First, regarding the background of cases, homecoming births outside Chiba Prefecture were not included; this may underestimate the incidence of invasive GBS disease. Second, the population aged 0 year on 1st April of the following year does not strictly indicate the number of live births in the previous year. Third, although this study was a prospective study, we could only examine 68 strains of 127 episodes for bacterial characteristics directly. No other strains were preserved in any of the hospitals where the GBS strain was isolated, and the remaining strains could not be collected and tested.

In conclusion, invasive GBS disease is a severe bacterial infection that occurs in the neonatal period and has a poor prognosis. Further prevention and treatment strategies are required, including a comprehensive appropriate maternal screening and the introduction of new vaccines and standard antimicrobial therapy guidelines.

## Data Availability

The data that support the findings of this study are available from the corresponding author, N.T., upon reasonable request.

## Supplementary material

For supplementary material accompanying this paper visit http://doi.org/10.1017/S0950268822001534. click here to view supplementary material

## References

[1] Edmond KM (2012) Group B streptococcal disease in infants aged younger than 3 months: systematic review and meta-analysis. The Lancet 379, 547–556. 10.1016/S0140-6736(11)61651-6 22226047

[2] Verani JR (2010) Prevention of perinatal group B streptococcal disease—revised guidelines from CDC, 2010. Morbidity and Mortality Weekly Report. Recommendations and Reports 59, 1–36. 21088663

[3] Pannaraj PS and Baker CJ (2019) Group B streptococcal infections. In Cherry JD, Harrison G, Kaplan S, Steinbach W and Hotez P (eds), Feigin and Cherry's Textbook of Pediatric Infectious Diseases. Philadelphia: Elsevier, pp. 823–834.

[4] Van Dyke MK (2009) Evaluation of universal antenatal screening for group B streptococcus. The New England Journal of Medicine 360, 2626–2636. 19535801 10.1056/NEJMoa0806820

[5] Melin P and Efstratiou A (2013) Group B streptococcal epidemiology and vaccine needs in developed countries. Vaccine 31, D31–D42. 23973345 10.1016/j.vaccine.2013.05.012

[6] Swamy GK (2020) Safety and immunogenicity of an investigational maternal trivalent group B streptococcus vaccine in pregnant women and their infants: results from a randomized placebo-controlled phase II trial. Vaccine 38, 6930–6940. 32883555 10.1016/j.vaccine.2020.08.056

[7] Chang B (2014) Characteristics of group B streptococcus isolated from infants with invasive infections: a population-based study in Japan. Japanese Journal of Infectious Diseases 67, 356–360. 25241685 10.7883/yoken.67.356

[8] Matsubara K (2017) Group B streptococcal disease in infants in the first year of life: a nationwide surveillance study in Japan, 2011–2015. Infection 45, 449–458. 28236250 10.1007/s15010-017-0995-2

[9] Shibata M (2022) Epidemiology of group B streptococcal disease in infants younger than 1 year in Japan: a nationwide surveillance study 2016–2020. European Journal of Clinical Microbiology and Infectious Diseases 41, 559–571. 35048277 10.1007/s10096-021-04396-y

[10] Chiba Prefecture. Population by age in Chiba Prefecture (in Japanese) Available at https://www.pref.chiba.lg.jp/toukei/toukeidata/nenreibetsu/index.html (Accessed 5 March 2022).

[11] Kong F (2002) Serotype identification of group B streptococci by PCR and sequencing. Journal of Clinical Microbiology 40, 216–226. 11773119 10.1128/JCM.40.1.216-226.2002 PMC120111

[12] Jones N (2003) Multilocus sequence typing system for group B streptococcus. Journal of Clinical Microbiology 41, 2530–2536. 12791877 10.1128/JCM.41.6.2530-2536.2003 PMC156480

[13] Pub MLST. Streptococcus agalactiae. Available at https://pubmlst.org/organisms/streptococcus-agalactiae (Accessed 5 March 2022).

[14] Clinical and Laboratory Standards Institute (2022) Performance Standards for Antimicrobial Susceptibility Testing, 32nd Edn. Clinical and Laboratory Standards Institute,Wayne, PA. Available at http://em100.edaptivedocs.net/GetDoc.aspx?doc=CLSI%20M100%20ED32:2022&scope=user (Accessed 5 March 2022).

[15] Japan Society of Obstetrics and Gynecology and Japan Association of Obstetricians and Gynecologists (2017) Guidelines for Obstetrical Practice, 2017 Edn. Japan Society of Obstetrics and Gynecology, Tokyo, pp. 341–344 (in Japanese).

[16] Nanduri SA (2019) Epidemiology of invasive early-onset and late-onset group B streptococcal disease in the United States, 2006 to 2015: multistate laboratory and population-based surveillance. JAMA Pediatrics 173, 224–233. 30640366 10.1001/jamapediatrics.2018.4826 PMC6439883

[17] Stoll BJ (2020) Early-onset neonatal sepsis 2015 to 2017, the rise of *Escherichia coli*, and the need for novel prevention strategies. JAMA Pediatrics 174, e200593. 32364598 10.1001/jamapediatrics.2020.0593 PMC7199167

[18] Suga S (2018) A nationwide population-based surveillance of invasive *Haemophilus influenzae* diseases in children after the introduction of the *Haemophilus influenzae* type b vaccine in Japan. Vaccine 36, 5678–5684. 30122645 10.1016/j.vaccine.2018.08.029

[19] Suga S (2015) Nationwide population-based surveillance of invasive pneumococcal disease in Japanese children: effects of the seven-valent pneumococcal conjugate vaccine. Vaccine 33, 6054–6060. 26235372 10.1016/j.vaccine.2015.07.069

[20] Lamagni T (2022) Assessing the added value of group B streptococcus maternal immunisation in preventing maternal infection and fetal harm: population surveillance study. BJOG: An International Journal of Obstetrics and Gynaecology 129, 233–240. 34324252 10.1111/1471-0528.16852 PMC9291181

[21] Kam KQ (2021) Serotype distribution and incidence of invasive early onset and late onset group B streptococcal disease amongst infants in Singapore. BMC Infectious Diseases 21, 1221. 34876053 10.1186/s12879-021-06891-1 PMC8650237

[22] Tazi A (2010) The surface protein HvgA mediates group B streptococcus hypervirulence and meningeal tropism in neonates. Journal of Experimental Medicine 207, 2313–2322. 20956545 10.1084/jem.20092594 PMC2964583

[23] Zimmermann P (2017) The controversial role of breast milk in GBS late-onset disease. The Journal of infection 74, S34–S40. 28646960 10.1016/S0163-4453(17)30189-5

[24] Miselli F (2022) Group B streptococcus late-onset neonatal disease. The Pediatric Infectious Disease Journal 41, e263–e266. 35446809 10.1097/INF.0000000000003517

[25] Kimura K (2008) First molecular characterization of group B streptococci with reduced penicillin susceptibility. Antimicrobial Agents and Chemotherapy 52, 2890–2897. 18490507 10.1128/AAC.00185-08 PMC2493108

[26] Moroi H (2019) Isolation of group B streptococcus with reduced *β*-lactam susceptibility from pregnant women. Emerging Microbes and Infections 8, 2–7. 30866792 10.1080/22221751.2018.1557987 PMC6455180

[27] Hata A (2020) Post-implementation survey to assess the strategies adopted to prevent neonatal group B streptococcal infections in Japan. Kansenshogakuzasshi 94, 654–661, (in Japanese with English abstract).

[28] Shibata M (2021) Relationship between intrapartum antibiotic prophylaxis and group B streptococcal colonization dynamics in Japanese mother-neonate pairs. Journal of Infection and Chemotherapy 27, 977–983. 33610482 10.1016/j.jiac.2021.02.006

[29] Bellais S (2012) Capsular switching in group B streptococcus CC17 hypervirulent clone: a future challenge for polysaccharide vaccine development. The Journal of Infectious Diseases 206, 1745–1752. 23002446 10.1093/infdis/jis605

