# Supplementary material for: Epidemiology and bacterial characteristics of invasive group B streptococcus disease: a population-based study in Japan in 2010–2020
Source: Epidemiol Infect. 2022 Oct 7;150:e184. doi: 10.1017/S0950268822001534 (PMC9987023; doi:10.1017/S0950268822001534)
Supplement: Supplementary file 1 [file S0950268822001534sup001.docx]

*Epidemiology and Infection*

**Epidemiology and bacterial characteristics of invasive group B streptococcus disease: A population-based study in Japan in 2010–2020**

Noriko Takeuchi^1^*, Bin Chang^2^, Kenichi Takeshita^3^, Sachiko Naito^3^, Yoshiko Takahashi^3^, Haruka Hishiki^3^, and Naruhiko Ishiwada^1^

Supplementary Materials

**Supplementary Table S1. Yearly changes in the types of invasive GBS disease in patients aged <1 year**

| Year | Number of cases | Incidence rate per 1,000 live births | EOD  (incidence rate per 1,000 live births) | LOD  (incidence rate per 1,000 live births) | LLOD  (incidence rate per 1,000 live births) | Unknown |
| --- | --- | --- | --- | --- | --- | --- |
|  |  |  |  |  |  |  |
| 2010 | 13 | 0.25 | 3 (0.06) | 7 (0.14) | 2 (0.04) | 1 |
| 2011 | 11 | 0.21 | 2 (0.04) | 5 (0.10) | 1 (0.02) | 3 |
| 2012 | 8 | 0.16 | 0 (0.00) | 7 (0.14) | 0 (0.00) | 1 |
| 2013 | 8 | 0.17 | 2 (0.04) | 6 (0.13) | 0 (0.00) | 0 |
| 2014 | 17 | 0.36 | 3 (0.06) | 10 (0.21) | 4 (0.08) | 0 |
| 2015 | 7* | 0.15 | 2 (0.04) | 4* (0.08) | 1 (0.02) | 0 |
| 2016 | 13* | 0.28 | 3 (0.06) | 9* (0.19) | 1 (0.02) | 0 |
| 2017 | 14 | 0.31 | 4 (0.09) | 9 (0.20) | 1 (0.02) | 0 |
| 2018 | 9* | 0.20 | 1 (0.02) | 8* (0.18) | 0 (0.00) | 0 |
| 2019 | 13* | 0.30 | 2 (0.04) | 11* (0.25) | 0 (0.00) | 0 |
| 2020 | 11 | 0.26 | 1 (0.04) | 10 (0.24) | 0 (0.00) | 0 |
| Total | 124 |  | 23 | 86 | 10 | 5 |

EOD, early-onset disease; LOD, late-onset disease; LLOD, late late-onset disease

*Recurrent cases were counted twice.

**Supplementary Table S2. Background information of patients with invasive GBS disease aged <1 year (n=120)**

|  |  | Total (%) | EOD | LOD | LLOD | Unknown |
| --- | --- | --- | --- | --- | --- | --- |
| Birth body weight | <1000 g  1000–1499 g  1500–2499 g  ≥2500 g  Unknown | 9 (7.5)  3 (2.5)  19 (15.8)  77 (64.2)  12 (10.0) | 1  1  5  16  2 | 5  1  13  58  5 | 2  1  2  3  2 | 1  0  1  0  3 |
| Gestational age | <28 weeks  28–36 weeks  37–41 weeks  Unknown | 11 (9.2)  16 (13.3)  81 (67.5)  12 (10.0) | 2  3  16  2 | 5  11  60  6 | 3  1  5  1 | 1  1  0  3 |
| Underlying disease | Yes  No  Unknown | 40 (33.3)  74 (61.7)  6 (5.0) | 6  15  2 | 19  55  3 | 5  4  1 | 5  0  0 |
| Clinical diagnoses* | Bacteraemia  Meningitis  Pneumonia  Others | 54 (43.5)  56 (45.2)  9 (7.26)  5 (4.2) | 8  10  5  0 | 41  41  0  4 | 4  5  0  1 | 1  0  4  0 |

EOD, early-onset disease; LOD, late-onset disease; LLOD, late late-onset disease

*Recurrent cases were counted twice.

**Supplementary Table S3. Maternal information of patients with invasive GBS disease aged <1 year (n=120)**

|  |  | Total (%) | EOD | LOD | LLOD | Unknown |
| --- | --- | --- | --- | --- | --- | --- |
| Mode of delivery |  |  |  |  |  |  |
|  | Caesarean section | 26 (21.7) | 2 | 20 | 4 | 0 |
|  | Vaginal delivery | 72 (60.0) | 19 | 50 | 3 | 0 |
|  | Unknown | 22 (18.3) | 2 | 12 | 3 | 5 |
| Maternal colonisation |  |  |  |  |  |  |
|  | Negative | 57 (47.5) | 17 | 36 | 4 | 0 |
|  | Positive | 17 (14.2) | 1 | 16 | 0 | 0 |
|  | Untested | 10 (8.3) | 2 | 7 | 1 | 0 |
|  | Unknown | 36 (30.0) | 3 | 23 | 5 | 5 |
| Prophylaxis of antimicrobials |  |  |  |  |  |  |
|  | Yes | 21 (17.5) | 2 | 19 | 1 | 0 |
|  | No | 63 (52.5) | 19 | 38 | 6 | 0 |
|  | Unknown | 36 (30.0) | 2 | 26 | 3 | 5 |
| Nutrition for infants |  |  |  |  |  |  |
|  | Breast milk | 18 (15.0) | 1 | 16 | 1 | 0 |
|  | Formula milk | 22 (18.3) | 3 | 18 | 1 | 0 |
|  | Mix | 45 (37.5) | 7 | 33 | 5 | 0 |
|  | Before the start of nutrition | 8 (6.7) | 7 | 1 | 0 | 0 |
|  | Breast milk and weaning food | 1 (0.8) | 0 | 0 | 1 | 0 |
|  | Unknown | 26 (21.7) | 5 | 13 | 3 | 5 |

EOD, early-onset disease; LOD, late-onset disease; LLOD, late late-onset disease
